# Key Labeling Technologies to Tackle Sizeable Problems in RNA Structural Biology

**DOI:** 10.3390/ijms9071214

**Published:** 2008-07-14

**Authors:** Kwaku T. Dayie

**Affiliations:** Lerner Research Institute, Department of Molecular Genetics and Center for Structural Biology, Cleveland Clinic, 9500 Euclid Avenue, Cleveland, OH 44195, USA

**Keywords:** dynamics, FRET, isotopic labeling, ligation, NMR, Raman, RNA dynamics and folding and structure, X-ray crystallography

## Abstract

The ability to adopt complex three-dimensional (3D) structures that can rapidly interconvert between multiple functional states (folding and dynamics) is vital for the proper functioning of RNAs. Consequently, RNA structure and dynamics necessarily determine their biological function. In the post-genomic era, it is clear that RNAs comprise a larger proportion (>50%) of the transcribed genome compared to proteins (≤2%). Yet the determination of the 3D structures of RNAs lags considerably behind those of proteins and to date there are even fewer investigations of dynamics in RNAs compared to proteins. Site specific incorporation of various structural and dynamic probes into nucleic acids would likely transform RNA structural biology. Therefore, various methods for introducing probes for structural, functional, and biotechnological applications are critically assessed here. These probes include stable isotopes such as ^2^H, ^13^C, ^15^N, and ^19^F. Incorporation of these probes using improved RNA ligation strategies promises to change the landscape of structural biology of supramacromolecules probed by biophysical tools such as nuclear magnetic resonance (NMR) spectroscopy, X-ray crystallography and Raman spectroscopy. Finally, some of the structural and dynamic problems that can be addressed using these technological advances are outlined.

## 1. Introduction

**Figure 1.**
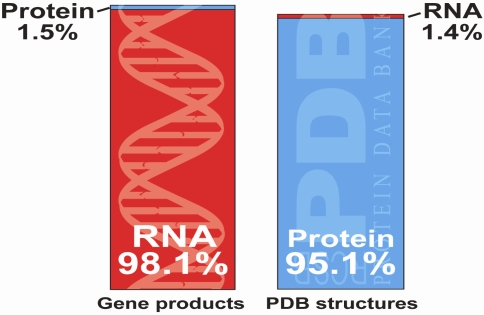
Inverse correlation between the genomic outputs of RNAs and proteins and the number of solved 3D structures deposited in the protein data bank.

Increasingly RNA molecules have taken center stage as key informational, structural, catalytic, and gene-regulatory molecules [1]. Contrary to the traditional view of the flow of genetic information wherein RNA is considered a minor intermediary for information transfer, RNAs have now been shown to be involved in a wide range of biological processes. In the cytoplasm, ribosomal RNAs catalyze and regulate protein synthesis [2–3], whereas in the nucleus small nuclear RNAs bound to proteins catalyze and regulate pre-RNA splicing [4–5]. In the nucleolus, small nucleolar RNAs (snoRNAs) form complexes with proteins to remodel the pre-ribosomal RNAs via methylation and pseudouridylation [6]. Finally, bacterial riboswitches influence transcription or translation by directly sensing metabolites or other environmental cues [7–8]. Unexpectedly, analysis of the human genome data reveals that only ~1.5% of the genome encodes proteins; about 60–70% of the genome represents non-protein coding transcribed RNA [9, 10]. Nevertheless, of the structures deposited in the Protein Data Bank, proteins constitute 95%, whereas RNAs account for less than 2% (Figure 1).

The calculation of 3D structures of RNA molecules by NMR and X-ray crystallography have, therefore, lagged substantially behind those of proteins. For NMR, this lag is partly because of extensive signal overlap and the rapid signal decay that occurs with increasing molecular weight. For X-ray crystallography, RNA structures have been difficult to solve partly because of RNA flexibility and the difficulty of finding suitable constructs that are able to diffract to high-resolution, as well as finding suitable heavy atom derivatives for phase determination. Recently, a number of emerging technologies make the prospects of tackling large supramacromolecular RNA complexes both probable and doable by various biophysical methods such as NMR, fluorescent resonance energy transfer (FRET), Raman, and X-ray crystallography. Several excellent reviews describe the technical details of NMR RNA structure calculation [11], advances in RNA NMR methodology [12–15], and RNA X-ray crystallography [16–19]. Here, we outline the various methods for preparing milligram quantities of natively folded RNA without denaturing and refolding, new methods for incorporating specific labels into RNA, efficient techniques for ligating RNAs together, and prospects for tackling large macromolecules by NMR spectroscopy and X-ray crystallography.

## 2. Synthesis and purification of milligram quantities of natively folded RNA for biophysical studies

NMR spectroscopic and X-ray crystallographic analyses require large milligram quantities of pure and functionally active materials. Chemical or biochemical methods allow synthesis of large quantities of RNA. While the chemical synthetic method affords site-specific incorporation of both standard and non-standard nucleobases into RNA, the labeled precursors are usually expensive and difficult to synthesize. Although chemical synthesis of RNA has improved considerably over the past decade, this method is highly inefficient beyond 50 nucleotides (nt) [20]. In this section both traditional methods and three new and improved methods for preparing natively folded RNAs are reviewed.

### 2.1 T7 RNA polymerase based transcription of RNA

The *in vitro* transcription with various DNA dependent polymerases such as SP6, T3, or T7 is a widely used enzymatic method [21–24] that overcomes some of the limitations of the chemical method. *In vitro* transcription by T7 polymerase has become the standard procedure for making RNA for many biophysical studies, however. Yet the T7 polymerase-based approach has at least three serious limitations. First, the widely used T7 consensus promoter (class III φ6.5 promoter) requires a guanosine as the first nucleotide for efficient initiation, as well as two or more consecutive guanosines at the first two to three nucleotide positions [22, 24]. This purine rich requirement at the 5′-end significantly restricts the types of RNA sequences that can be synthesized efficiently. Second, repeated abortive transcription initiations result in a high degree of 5′-end heterogeneity [25, 26]. Third, T7 RNA polymerase adds one or more nontemplated nucleotides at the 3′-end of the nascent RNA transcript to produce fragments that are longer than expected. These N+1 and N+2 products can be produced in equimolar or higher amounts than the desired product [22, 27]. Previous reviews describe the advantages and limitations of using DNA templates of various lengths for transcription, common RNA refolding schemes, and the resolution of various purification methods at the nucleotide level [22, 28–29]. Here those limitations that have been addressed recently are detailed.

Several approaches have been proposed to circumvent these heterogeneity problems. The 3′-end heterogeneity problem can largely be overcome using *cis*- or *trans*-acting ribozymes to cleave at specific positions to leave homogenous length RNA [30, 31]. Another method makes use of non-polar nucleoside analogues (4-methylindole β-deoxy nucleoside) [32] or DNA templates modified in the two terminal nucleotide positions with 2′-methyoxyribose sugars to substantially reduce the 3′-end heterogeneity [33]. Similarly, the T7 class II φ2.5 promoters that initiate transcription with adenosine triphosphates (ATP) and ATP analogues have been shown to possess superior 5′-homogeneity compared to the GTP-initiating T7 class III φ6.5 promoters [34]. The use of cytidine at the beginning of the template strand together with 2′-methoxyribose is adequate to improve yield and reduce heterogeneities [35]. In our experience, transcription optimization using a sparse-matrix approach is necessary for optimal yield. For example, the concentrations of Mg^2+^, NTP, and the T7 RNA polymerase can be varied systematically to achieve an optimum yield of the full length product that can reach up to 5 A_260_ units/ml of transcription [29, 35].

An implicit assumption in all discussions about making RNAs is that T7 polymerase-based transcribed RNAs are correctly and homogenously folded. To evaluate this possibility before investing substantial effort and time into a non-denaturing purification scheme, it is important to show that the RNA adopts the structure of interest at the concentrations needed for the biophysical studies. Access to appropriate biochemical and functional assays are useful in this regard.

A completely different *in vivo* method, that may alleviate some of these concerns, was recently illustrated by Ponchon and Dardel [36]. The authors described a generic approach to express and purify structured RNAs in *Escherichia coli* (*E.coli*) using tRNA-RNA fusions, similar to glutathione S-transferase fusions used for protein expression. This tRNA fusion serves to protect the desired RNA from cellular RNAse degradation of [36]. It remains to be seen how general this approach will be for synthesizing large RNAs with complex domains or RNAs with significant single-stranded regions.

### 2.2 Native purification of RNA for biophysical studies

**Figure 2.**
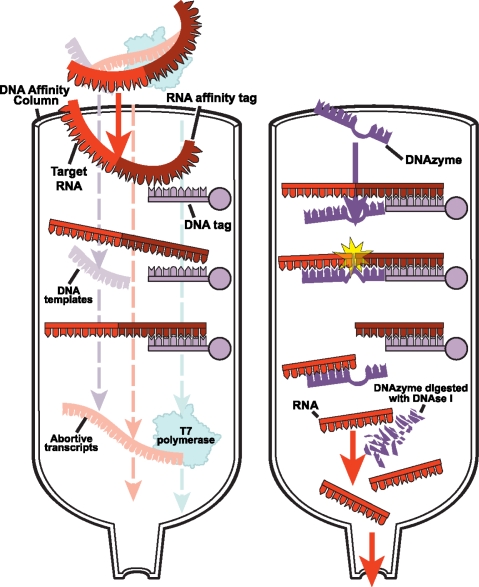
Schematic of native purification of RNA using self-cleaving DNAzyme affinity chromatography. The DNAzyme has a core structure of 15 nt and two flanking arms of 7–13 nt which can hybridize to the target RNA, and 15 of the 20 nt of the DNA affinity tag is complementary to the target RNA.

**Figure 3.**
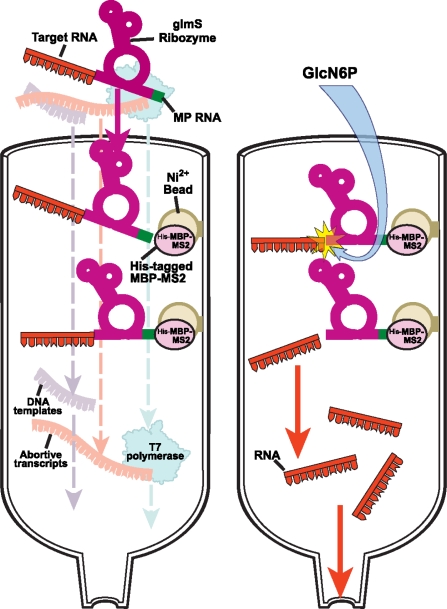
Schematic of native purification of RNA using self-cleaving *glmS* Ribozyme (~140 nt) and Ni^2+^ affinity chromatography. The MS2 coat protein binding RNA stem-loops is 43 nt.

After *in vitro* or *vivo* synthesis, the target RNA is purified away from unwanted side-products and unused reactants using traditional denaturing or non-denaturing methods. The traditional purification methods include anion-exchange high-performance liquid chromatography (HPLC) [37–39], ion-pairreversed phase HPLC [40–42], and denaturing polyacrylamide gel electrophoresis (PAGE) [28, 43–44]. For short RNAs (<20 nt) the HPLC methods can be used to produce homogenous-length RNAs rapidly, and for medium-sized RNAs (<50 nt) the preparative denaturing PAGE method is a robust and well-established method of choice. Nonetheless, both HPLC and PAGE methods denature the RNA; then the RNA is refolded following purification. Furthermore, the step preceding the gel loading requires phenol-chloroform extraction, which may result in sample loss and RNA aggregation. Finally, a 20 ml transcription reaction requires at least four conventional 40 × 60 × 0.3 cm^3^ PAGE gel runs that are tedious and time-consuming. Three promising approaches to avoid the denaturing steps of preparation and to speed up the purification process are outlined below (Figures 2 and 3).

#### 2.2.1 DNA affinity column chromatography for native purification of RNA

Cheong and coworkers [45] used trans-acting DNA enzymes (DNAzymes) and DNA affinity column chromatography to purify milligram quantities of RNA in 3 days (Figure 2). The affinity column was synthesized chemically using 1 gram of Oligo Affinity support from Glen Research. A tag sequence appended to the 3′-end of the RNA binds a complementary DNA sequence on the column allowing the abortive transcripts, DNA template, T7 polymerase, and unused NTPs to be washed off the column. The bound RNA is eluted under low salt conditions and then digested with the DNAzyme to produce homogenous 3′-ends. The affinity column further separates this mixture by retaining the 3′-affinity tag on the column, but the target RNA or the DNAzyme flows through. Finally, the DNAzyme is digested with DNAse I followed by size exclusion chromatography. The authors do not indicate the attainable yield. However, it is likely the ratio of the final product to the initial transcript is substantially less than 50%. The approach solves the 3′-heterogeneity problem, and it obviates the need for a denaturing preparation. The method, however, does not solve the 5′-heterogeneity problem. But this problem can be easily rectified using other DNAzymes or a hammerhead ribozyme construct at the 5′-end. Such an improved affinity capture method could make it more high-throughput and broadly adopted by the biophysical community in the future.

#### 2.2.2 Protein-RNA affinity column chromatography for native purification of RNA

Concurrently, the groups of Batey and Kieft also proposed a general method for purifying RNA under native conditions within 24 hours. They took advantage of an RNA-protein interaction to immobilize the transcribed RNA onto an affinity column and an imidazole activated hepatitis delta virus (HDV) to cleave the desired product off the column [46]. Their two domain affinity tag contained a cytidine-to-uridine (C75U) variant of the HDV ribozyme and a stem loop RNA motif from a thermostable signal recognition particle (SRP) that binds tightly and specifically to the SRP protein, the Ffh-M domain protein. The transcribed RNA is run over an affiGel affinity column to which the Fth protein has been immobilized. Abortive transcripts, DNA template, T7 polymerase and other unwanted products come off the column while the full-length RNA remains on the column. Addition of high concentration imidazole, ~200 mM, cleaves the target RNA and enables a clean elution of the RNA. Unfortunately, this earlier version had many technical problems, which have been addressed subsequently [47]. First, the Ffh-M domain protein precipitates in low salt concentration during cation exchange chromatography and the column coupling steps. Second, the columns become ineffective after repeated use. Third, the use of very high imidazole concentrations for the cleavage step on the column significantly degrades the RNA, and for certain RNA sequences cleavage remains incomplete even after prolonged incubation [47].

To improve on the older design, Batey and Kieft now replaced the problematic Fth protein with a his-tagged form of the maltose binding protein (MBP)-MS2 coat fusion protein that can bind to the widely used Ni^2+^ [48] affinity columns (Figure 3). They substituted the mutant HDV ribozyme with the *glmS* ribozyme that can be activated by glucosamine-6-phosphate (GlcN6P) at low concentration of ~1 mM [49]. In place of the Fth-M RNA binding site, they inserted two copies of the MS2 coat protein binding RNA stem-loops upstream of the *glmS* ribozyme.

With this new scheme, small and large scale purifications can be conveniently carried out. For small-scale RNA synthesis, the entire *in vitro* transcription reaction is pre-incubated with the MBP-MS2 protein and the resulting complex is applied to a Qiagen Ni^2+^ spin column. For large scale preparation, the complex is applied to a Qiagen Ni^2+^-agarose resin packed into a disposal column. After washing off incomplete transcripts and unused NTPs, the product RNA is eluted with 1mM GlcN6P (Figure 3). To prevent substantial amount of background cleavage in low concentrations of Tris (~10mM), the authors recommend the use of K^+^-HEPES.

This method has a few disadvantages. First, it can be further improved by incorporating schemes to suppress 5′-end heterogeneity as discussed above. From hydroxyl radical footprinting experiments, Hampel and Tinsley showed that the first four unpaired nucleotides upstream from the cleavage site of the *glmS* ribozyme are protected [50]. Thus, a minimum structural requirement for cleavage might be four bases from the 5′ cut site. Finally, the cleavage by *glmS* ribozyme leaves an adenine at the 3′-end of the RNA product. So far as it does not affect function, this extra adenine is small price to pay for obtaining natively-folded RNA samples.

#### 2.2.3 Size exclusion chromatography for native purification of RNA

Alternatively, Puglisi and colleagues have employed size-exclusion chromatography to purify large RNAs in a day [51, 52]. A recent publication from this group provides details of the practical applications of this method [53]. In their method, after quenching with EDTA, the reaction mixture is subjected to phenol-chloroform extraction to remove the restriction enzyme carried over from the DNA template digestion and T7 RNA polymerase. A desalting column then removes any remaining phenol and NTPs. The resulting aqueous phase is applied to either a Superdex 200 size-exclusion column for RNAs between 120 and 400 nt or to Superdex 75 for RNAs between 10 and 150 nt.

A drawback of this method is the phenol-chloroform extraction step, which can result in RNA aggregation, sample loss and inability to resolve RNAs with heterogenous ends. The method, nevertheless, should become very rapidly adopted because of the ease of operation, the ability to separate monomeric and higher-order conformations, and the facile removable of water soluble acrylamide contaminants that plague polyacrylamide gel electrophoresis methods.

In sum, the methods of affinity and size-exclusion chromatography, unlike PAGE and HPLC that denature the RNAs, should prove very useful for preparing natively folded RNAs for functional studies and biophysical characterization.

## 3. Isotopic labeling of RNA for biophysical studies

Access to ^15^N and/or ^13^C labeled proteins, which were enriched from <1% to close to 100% ^15^N/^13^C, led to the development of multidimensional heteronuclear NMR experiments, and the transformation of solution NMR structure determination of proteins. Similar advances in methods for synthesizing isotopically labeled RNA [43–44, 54–55], which immediately followed the protein advance [56], inspired development of new heteronuclear NMR experiments [13–15, 57–59].

**Figure 4.**
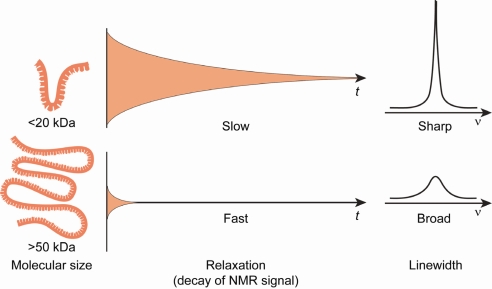
Small molecules in solution tumble rapidly and the corresponding NMR signal decays slowly and has narrow linewidth, whereas large molecules tumble slowly in solution and the resulting NMR signal decays rapidly and has broad lines; these broad lines have diminished signal-to-noise compared to the narrow lines.

Even so, it soon became clear that ^13^C and/or ^15^N labeling was very useful for medium sized RNAs (≤30 nt) only. As the size of the RNA increased, two challenges facing RNA structural biology become even more acute: extensive spectral crowding and increased resonance linewidths. The spectral overlap problem is acute for RNA because the non-exchangeable ribose protons (H2′-H5′, H5″) resonate in a very narrow chemical shift range of 1 ppm. The number of protons within this spectral window scales linearly with the number of nucleotides in an RNA molecule without a change in spectral width. As a result, the spectral overlap in RNA molecules >40 nt is so severe that assignment of the individual resonance peak positions is extremely challenging. Additionally, line broadening results from faster relaxation of the magnetization, and this effect increases proportionally with molecular weight (Figure 4). Because of all these problems, alternate labeling strategies have become necessary. In this section, the procedures developed for uniform and alternate site-specific ^13^C and/or ^15^N labeling, ^2^H labeling, and ^19^F labeling are outlined.

### 3.1 ^15^N and ^13^C isotopic labeling of RNA for biophysical studies

**Figure 5.**
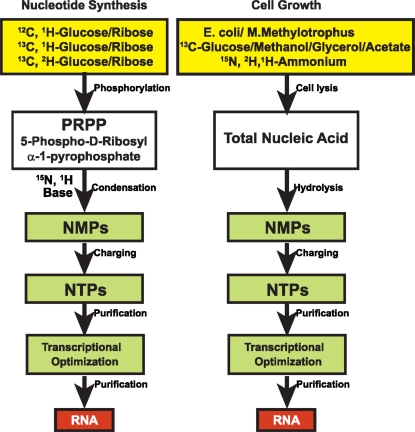
Strategies to prepare isotopically enriched RNA using labeled carbon and nitrogen sources.

To overcome the overlap and relaxation problems, RNAs can be labeled uniformly, randomly, or site-specifically with stable isotopes (Figure 5). The preferred labeling pattern is accomplished by growing either *E.coli* or *Methylobacterium* on defined chemical media and harvesting the total cellular RNA. Enzymatic digestion of the cellular RNA to nucleotide monophosphates (NMPs) and rephosphorylation of the NMPs yields the nucleotide triphosphates (NTPs) needed for *in vitro* RNA transcription.

In the past, ^13^C or ^13^C/^15^N labeled nucleotides were produced by growing the organism *Methylophilus methylotrophus (M. methylotrophus)* [44] or *Methylobacterium extorquens* [55] on ^13^C-methanol for three reasons. First, methanol was a very economical source of ^13^C label at the time this method was developed; that is, the price difference between ^13^C labeled methanol and glucose was approximately 600% [44]. These prices are now comparable. Second, a maximum yield of RNA was achievable for every gram input of labeled methanol. *M. methylotrophus* grows rapidly on methanol with a doubling time of 1.9 hours, and the percentage of dry cell weight that is RNA increases with a decrease in doubling time. Third, the use of *methylotrophs* enables partial and random incorporation of both ^13^C and ^2^H [55, 60].

Compared to *E.coli*, the use of methylophiles has four disadvantages. First, isotopic scrambling during biosynthesis prevents incorporation of site-specific isotopic labels using methanol. Second, methylophiles have significantly lower ribonucleotide content per gram of cells. Specifically, the yield of NMPs from *M. methylotrophus* is 42% of that for *E.coli* [44]. Third, the growth of *M. methylotrophus* is not as easily controlled as that of *E.coli*. And fourth, methylophiles are less accessible to many investigators familiar with use of *E.coli* for making proteins [44]. For all these reasons, *E.coli* is likely going to be favored over methylobacteria for labeling RNA.

### 3.2 Alternate site-specific ^13^C isotopic labeling of RNA with different E.coli strains

For RNAs 30 nt or smaller the resolution afforded by uniform labeling can be spectacular, but for larger RNAs alternative labeling strategies are needed to overcome both the crowding and increased linewidth problem. To address some of these limitations, various groups have developed single and multiple residue type labeling with some limited success [57, 61–62].

A promising option to alleviate the overlap problem is to grow various strains of *E.coli* on different combinations of carbon and nitrogen sources to achieve alternate site-specific labeling. Wild-type *E.coli* can be grown on ^13^C-sodium acetate as the sole carbon source, but the distribution of ^13^C atoms within the nucleotides that results is not uniform [63]. In other words, depending on which carbon site is labeled in the acetate carbon source, different parts of the ribonucleotide are labeled. For example, growing wild-type *E.coli* on ^13^CH_3_COONa as the only carbon source leads to >90% labeling efficiency of not only the carbon positions of C5 and C6 within pyrimidine rings but also the carbon positions of C2, C6 and C8 within purine rings. Moreover, all the carbon positions, except the C3′ position, within the ribose ring are labeled at 80–90% efficiency. Of great interest, the resulting NTPs are highly ^13^C-enriched at all the positions useful for proton-detected NMR experiments. This labeling pattern, however, does not alleviate the problem of strong ^13^C-^13^C J-coupling for either the C5 and C6 positions within pyrimidine ring or for all the carbon positions within the ribose ring. As a second example, growing wild-type *E.coli* on CH_3_^13^COONa as the only carbon source leads to >90% labeling efficiency of the carbon positions of C2 and C4 within pyrimidine rings and C4 and C6 within purine rings. In this case, the resulting NTPs are highly ^13^C-enriched at positions not useful for proton-detected NMR, but may be beneficial for other biophysical applications such as Raman spectroscopy or Mass spectrometry.

The growth of *E.coli* on ^13^C-formate and unlabeled glucose, on the other hand, leads to site-specific incorporation of the ^13^C label at the C8 position of purines with >85% efficiency [59]. Selective labeling of the C8 carbon in purines removes the ^13^C-^13^C J-couplings to C4 and C5 carbons. Besides, this label eliminates the overlap of the C8 and C4/C5/C6 carbon atoms that resonate in similar spectral regions. Since both coupling and overlap problems interfere with accurately analyzing T_2_-CPMG (Carl-Purcell-Meiboom-Gill) data used for relaxation measurements, this C8 specific labeling strategy is advantageous for the analysis of ^13^C relaxation data.

Hoogstraten and his group adopted an alternate-site isotopic labeling scheme for incorporating ^13^C label into specific ribose carbon positions using mutant *E.coli* strains [64]. By growing an *E.coli* strain deficient in the trichloroacetic acid cycle (DL323, available from the Yale Coli Genetic Stock Center, #7538) on 2-^13^C glycerol, a mixture of partially labeled 1′, 4′-^13^C (55%) and 2′, 4′-^13^C (30%) ribose isotopomers are obtained. Likewise, the DL323 *E.coli* strain grown on 1, 3-^13^C-glycerol yields ~90% ^13^C label at only the C5 position of CMP, and when grown on 2-^13^C-glycerol gives ~90% ^13^C label at only the C6 position of CMP. Another *E.coli* strain deficient in the glucose-6-phosphate dehydrogenase pathway, strain K10-1516 (available from the Yale Coli Genetic Stock Center, #4858), grown on 2-^13^C-glycerol gives superior labeling mostly at the C2′ (~98%) and C4′ (>95%) ribose positions. As an added benefit, this latter labeling scheme gives 50% labeling efficiency for both C5 and C6 carbon sites in the purine ring. Overall, the K10-1516 strain is the most attractive for selectively labeling at C2′ and C4′ and C5/C6 positions at >80% efficiency. These labels largely remove the ^13^C-^13^C interference effects. Hoogstraten and Johnson recently described how these labels effectively remove these couplings [65]. As a result, they provide an ideal two-spin system for NMR relaxation studies. Finally, their use in labeled RNA should enable analysis of RNA spectral regions that are notoriously overcrowded without resorting to complex NMR experiments [35, 66].

### 3.3 Deuterium (^2^H) isotopic labeling of RNA for biophysical studies

**Figure 6.**
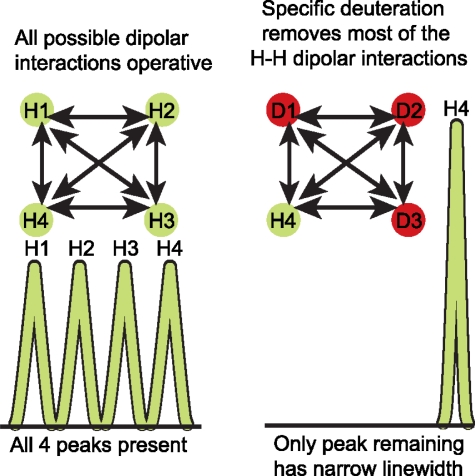
By eliminating competing relaxation pathways among dipolar coupled protons, deuteration leads to reduced linewidths, increased signal-to-noise and reduced chemical shift overlap in NMR spectra.

Of the many isotopic labeling approaches proposed to deal with both the overlap and the rapid relaxation problem, use of deuterium labeling remains the most attractive for improving spectral resolution and combating the relaxation problem [67–73]. Deuterium labeling of RNA functional groups increases the level of deuterium from the natural abundance value of 0.015% to >90%. Deuterium has a gyromagnetic ratio that is ~6.5 times smaller than that of hydrogen. Therefore, relative to protons, the relaxation rates of the deuterium spin attached to a heteroatom such as ^13^C or ^15^N is scaled down by 2% [(γ_D_/γ_H_)^2^ ~ 0.02]. In addition, the energy of transition for deuterium is very different from that of hydrogen such that the deuterium resonances are absent in a proton NMR spectral region. Of the two types of deuterium labeling, random fractional deuteration, wherein organisms are grown on partially deuterated carbon sources in a partial D_2_O solvent mixture, is not as effective for RNA NMR as it is for protein NMR. In contrast, specific deuteration, where ^2^H is placed in a specific location of a macromolecule, results in homogenous labeled molecules without uniform reduction in signal intensity (Figure 6). This makes the specific deuteration approach very attractive.

### 3.4 ^2^H, ^13^C, and ^19^F isotopic labeling of RNA for biophysical studies

By using various combinations of uniformly or specifically labeled glucose, the Williamson group showed that the ribose ring can be differentially labeled [70–71, 74–77]. Several applications have been reported [70–71, 75–77]. Because of the generality of this approach, we next outline this synthetic strategy in some detail.

**Figure 7.**
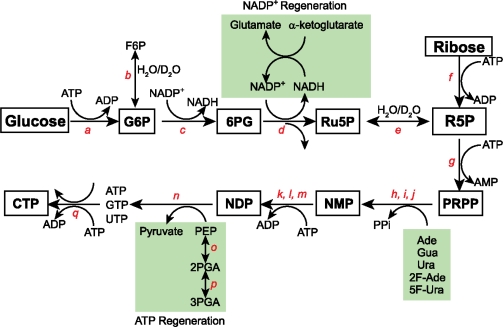
Schematic of the enzymatic reaction to convert (un)labeled ribose or glucose to the four NTPs needed for RNA synthesis. Most of these enzymes are available from Sigma-Aldrich unless indicated otherwise. The enzymes (only those not commercially available are indicated as *NA*) involved are: (a) hexokinase (E.C.2.7.1.1), (b) glucose-6-phosphate isomerase (E.C.5.3.1.9), (c) glucose-6-phosphate dehydrogenase (E.C.1.1.1.49), (d) 6-phosphogluconate dehydrogenase (E.C.1.1.1.44), (e) ribose-5-phosphate isomerase (E.C.5.3.1.6), (f) ribokinase (E.C.2.7.1.15; *NA*), (g) PRPP synthetase (E.C.2.7.6.1; *NA*), (h) adenine phosphoribosyltransferase (E.C.2.4.2.7; *NA*), (i) xanthine-guanine phosphoribosyltransferase (EC.2.4.2.22; *NA*), (j) uracil phosphoribosyltransferase (E.C.2.4.2.9; *NA*), (k) myokinase or adenylate kinase (E.C.2.7.4.3), (l) guanylate kinase (E.C.2.7.4.8), (m), nucleoside monophosphate kinase (E.C.2.7.4.4; Roche), (n) pyruvate kinase (E.C.2.7.1.40), (o) enolase (E.C.4.2.1.11), (p) 3-phosphoglycerate mutase (E.C.5.4.2.1; USB), and (q) CTP synthetase (E.C.6.3.4.2; *NA*). Note that for the last step of making CTP from UTP, L-glutamine or NH_3_ can be use for the amination reaction. In addition, the reductive amination of α-ketoglutarate is catalyzed by glutamic dehydrogenase (E.C.1.4.1.3).

In the enzymatic synthetic strategy adopted by the Williamson group (Figure 7), glucose is converted into the activated 5-phospho-D-ribosyl-α-1-pyrophosphate (PRPP) using the enzymes of the pentose phosphate pathway. This activated PRPP is converted into the four nucleoside triphosphates (ATP, GTP, UTP; CTP is prepared from UTP in a separate reaction) using phosphoribosyltransferases. This efficient enzymatic synthesis begins with the C6-phosphorylation of glucose by hexokinase to give glucose-6-phosphate (G6P). This C1 aldose (G6P) is oxidized by glucose-6-phosphate dehydrogenase to 6-phosphogluconate (6PG), and in turn 6-phosphogluconate dehydrogenase decarboxylates this β-keto carboxylic acid (6PG) to ribulose-5-phosphate (Ru5P). Ru5P is then isomerized to ribose-5-phosphate (R5P) by ribose-5-phosphate isomerase. Alternatively, ribokinase can directly phosphorylate the C1 position of ribose to generate R5P [76]. R5P, from either glucose or ribose, is then converted to PRPP by the phosphorylation of R5P'sC1 position by PRPP synthetase.

At this point in the synthesis a variety of labeled or unlabeled free bases can be directly coupled to PRPP. These condensation reactions are catalyzed by phosphoribosyltransferases specific for any of the bases, or modified forms of the bases (adenine, guanine, uracil, and their derivatives), to form nucleotide monophosphates (AMP, GMP, and UMP). The NMPs are subsequently converted to the nucleotide triphosphates by the sequential action of adenylate kinase or myokinase, guanylate kinase, nucleoside monophosphate kinase, and pyruvate kinase. The five phosphates and two oxidizing equivalents required for this synthesis are provided by ATP and NADP^+^. The ATP consumed during the reaction is regenerated *in situ* from phosphoenolpyruvate (PEP) by the action of enolase and pyruvate kinase. 3-phosphoglycerate mutase converts the relatively inexpensive D-(−)-3-phosphoglyceric acid (3PGA) to D-(+)-2-phosphoglyceric acid (2PGA), enolase forms PEP from 2PGA, and then pyruvate kinase catalyses the conversion of ADP to ATP [78–79]. The NADP^+^ required for oxidizing G6P and 6PG is regenerated by reductive amination of α-ketoglutarate with NADPH and ammonia catalyzed by glutamic dehydrogenase [80].

A key advantage of this labeling scheme is the ability to mix and match various combinations of labeled glucose or ribose with labeled bases. Again, this enzymatic strategy allows for the facile exchange of the C1′ and C2′ ribose positions with either ^1^H or ^2^H. Glucose-6-phosphate isomerase catalyzes the exchange of the solvent hydrogen or deuterium atoms with the atom attached to the C2 position of G6P. Specifically, if ^2^H is desired at the H2′ position then the reaction is better carried out in D_2_O and if ^1^H is desired then the reaction must be conducted in H_2_O. Thus, omission of glucose-6-phosphate isomerase leads to selective protonation or deuteration of the C2′ ribose position. As an unavoidable, but desirable, side effect of the pentose phosphate pathway, the proton destined to become the H2′ proton comes from the solvent milieu. R5P isomerase is able to remove a solvent molecule originally placed at the C1 position of Ru5P to its C2 position to form R5P. The combined action of R5P isomerase and G6P isomerase enables a combinatorial labeling of the C1′ and C2′ positions with either ^1^H or ^2^H.

Using this general strategy, the Williamson group has produced several isotopic labeling patterns [70–71, 75–77, 81–82]. Starting from fully deuterated [1, 2, 3, 4, 5, 6, 6-^2^H_7_]-d-glucose and conducting the reaction in D_2_O without G6P isomerase, 84% of the starting glucose is converted to the four NTPs with the ribose fully deuterated, a label called d_6_-NTP. By repeating this reaction in water and in the presence of G6P isomerase, the H1′ and H2′ ribose positions are selectively protonated in a background of deuterated ribose. This label is called d_4_-NTP. By carrying out the reaction in H_2_O with G6P isomerase using completely deuterated and uniformly ^13^C labeled [1, 2, 3, 4, 5, 6, 6-^2^H_7_-1, 2, 3, 4, 5, 6-^13^C]-d-glucose, d_4_-^13^C-ribose NTPs are easily prepared [70–71]. By simply omitting G6P isomerase and carrying out the reaction in H_2_O using completely deuterated and uniformly ^13^C labeled glucose shown above, d_5_-^13^C-ribose NTPs are easily prepared.

All five isotopic labeled patterns are valuable for NMR spectroscopic analysis of RNA structure and dynamics. The d_4_-^13^C-ribose NTPs are valuable for preparing RNA uniformly ^13^C-labeled in the ribose ring with H3′, H4′, H5′/H5″ protons specifically replaced with deuterons [71]. This labeling pattern simplifies the carbon heteronuclear single quantum correlation (HSQC) spectrum by removing the H3′-C3′ resonances that normally obscure the H2′-C2′ resonances. Furthermore, this d_4_ labeling pattern retains most of the useful structural information: the H1′-H2′ scalar coupling that reports on ribose pucker; base to H1′ NOE that provides the glycosidic torsion; and exchangeable protons that provide base pairing information [71]. These set of nucleotides (d_4_, d_4_-^13^C, d_6_, and combinations of d_4_ and d_6_) are important for NMR because their incorporation into an RNA molecule is valuable for recording NMR experiments that are easy to interpret and analyze. For example, the nuclear Overhauser spectroscopy (NOESY) experiment of such a sample has very interpretable base to ribose proton region that is difficult to assign otherwise [70, 75]. RNAs made with the d_5_-^13^C-ribose pattern have only the 2′-position protonated, and all the other ribose protons are specifically replaced with deuterons as described in detail next.

The research group of Kay, in collaboration with the Williamson group, showed that d_5_-^13^C-NTPs can be useful for relaxation analysis [77]. Here, the ribose sugar is not only protonated specifically at the 2′-position and all other positions remain deuterated, but also all the sugar carbons are ^13^C-labeled and the pyrimidine bases were ^2^H-labeld at the C5 position. This labeling strategy represents a significant improvement over partial (50%) deuteration within a fully ~100% ^13^C-enriched background because the latter selects ~25% signals with concomitant compromise in sensitivity. With close to 100% ^2^H label at C1′, C3′, C4′, and C5′ carbon sites, the authors probed the backbone dynamics of most of the deuterated sites in HIV-2 TAR RNA [77].

As another excellent example of the versatility of this enzymatic approach, Fluorine-19 (^19^F) nucleus can be incorporated into RNA bases [81–82]. This is apt because ^19^F can provide a wealth of geometrical and dynamical information about RNA molecules. ^19^F is not endogenously expressed in biological molecules making it a valuable probe, it has 100% natural abundance and a gyromagnetic ratio comparable to that of a proton (γ_F_~0.94γ_H_) which means it possesses an intrinsic sensitivity almost as high as that of a proton. Importantly, ^19^F also offers chemical shift dispersion (~900 ppm) ~50 times larger than that obtainable from protons. Recently, the research groups of Henning and Williamson proposed a facile T7 RNA polymerase based incorporation of 5-Fluoro Cytidine, 5-Fluoro Uracil and 2-Fluoro Adenine into a 30 nt HIV-2 TAR RNA construct [81–82].

On the whole, the Williamson approach is extremely flexible in decoupling ribose from base labeling. The commercial availability of all these labeling patterns (from Cambridge Isotope Labs and Cassia LLC) and further development of other labels will greatly facilitate structure determination of large RNAs especially using ligation strategies (See below).

Two limitations of this enzymatic approach must be overcome to make this method widely adopted by the biophysics community. First, the price for bases with various labels is high. Uracil uniformly labeled with ^13^C and ^15^N (U-13C4, 99%; U-15N2, 98%) currently lists at $750 for 0.1 g from Cambridge Isotope Laboratories. Incorporating the commercially available bases into the enzymatic labeling scheme would, therefore, be a costly method. Second, 7 out of the 18 enzymes required for synthesis are not commercially available and must be produced from over-expressing *E.coli* strains. Of these seven, only ribokinase and APRT have robust activities of 350–700 U (U, the activity of the purified protein from a 1 L culture, is defined as the amount of the enzyme that catalyses the reaction of 1 μmol of substrate per minute). The other five are only moderately over-expressed with activities of 28–40 U, making the enzyme preparation labor intensive [70, 74, 76]. By finding cheaper avenues to make labeled bases and constructs that highly over-express several of the commercially unavailable enzymes, the cost and labor required to synthesize these isotopically labeled NTPs could be significantly reduced.

### 3.5 Phasing strategies in RNA crystallography

X-ray crystallography is the other powerful approach for 3D structure determination of macromolecules. A substantial barrier for RNA structure determination by X-ray crystallography is heavy atom derivatization for phase determination [17, 83]. The phase problem arises because an X-ray diffraction experiment yields intensities without the phase information needed for reconstructing the original electron density.

In the past RNA crystallization tags have been used to address this problem. One example is the “soak and pray” method, wherein heavy atoms are soaked into the crystal to help with anomalous scattering [84–86]. Others involve RNA covalent modification with halogens or other heavy atoms [87–89], and introduction of protein binding sites into the RNA constructs [90–91]. Here two recent methods that appear promising to resolve the RNA crystallography phasing problem are presented.

#### 3.5.1 Selenium (Se) labeling of RNA for biophysical studies

**Figure 8.**
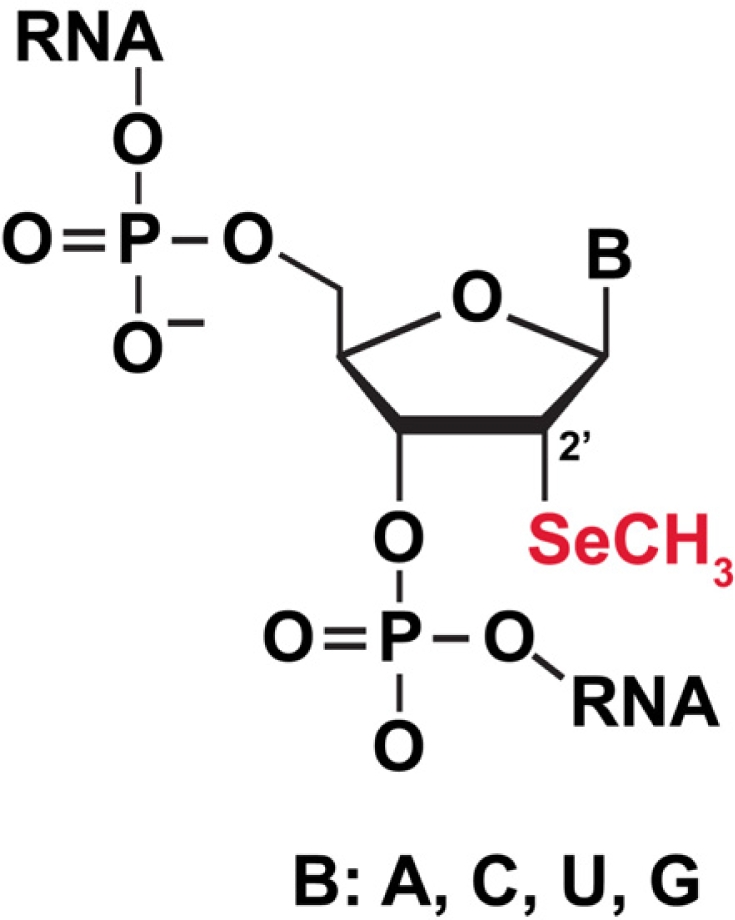
Incorporation of 2′-methylseleno group for derivatizing RNA for crystallization.

One method, multiple anomalous dispersion (MAD), aims to solve the phase problem by using intrinsic or exogenous anomalous scatterers. Incorporation of Selenium, an attractive element for derivatization of biomolecules, into proteins led to an explosive growth of new protein crystal structures. In contrast to proteins, derivatizing nucleic acids for X-ray crystallography has lagged behind. Recently the groups of Egli, Huang, and Micura have developed a selenium derivatization strategy for nucleic acids (Huang and Sheng provide greater detail in this series, [92]). Two positions within the nucleic acids have been derivatized, either by replacement of the 2′-OH with a 2′-methylseleno group (Figure 8) or replacement of the non-bridging phosphorus atom in the phosphodiester backbone. For instance, Huang and colleagues recently derivatized the hammerhead ribozyme with 5′-(α-P-seleno) NTPs using T7 RNA polymerase that readily accepts the *Sp* diasteromer to synthesize RNA [93]. The function of ribozymes depends on the diasteromer in the phosphate backbone, so it remains to be seen how general this approach will be.

#### 3.5.2 GU platforms for direct soaking of divalent metals for RNA for biophysical studies

Given the current limitations of chemical modifications of RNA for phase determinations, the research groups of Batey and Kieft proposed a direct soaking method. GU wobble pair motifs are intentionally engineered into seven variants of the SRP RNA to provide heavy atom binding sites with high occupancy and low B factors [94]. They demonstrated they could successfully solve the structure of three novel RNA sequences that had been refractory to crystallization using other methods [94]. How general is this approach? It is likely to be useful on a case-by-case basis. What is clear is that there are now a number of different approaches to solving the phase problem in RNA X-ray crystallography. These are welcome additions to previous efforts to solving the phase problem as discussed earlier.

## 4. Improved methods for ligating large RNAs for biophysical studies

Solving high-resolution structures of RNA molecules by X-ray crystallography and NMR spectroscopy is central to obtaining insights into fundamental biological processes. As discussed earlier, major hurdles for NMR structural analyses are crowding and signal decay, and for X-ray crystallographic analyses these are preparation of suitable heavy atom derivatives that are essential for calculation of crystallographic phases and generation of an electron density map. While improved methods for chemical synthesis of RNA exist, generating larger RNAs that are site-specifically modified with probes to help overcome some of these obstacles is more difficult. A very promising approach is joining RNA pieces together, which can be accomplished in at least four different ways: by chemical ligation, by DNA splinted DNA T4 ligation, by T4 RNA ligation, or by deoxyribozyme mediated ligation. In this section, the salient features of each method, as well as their advantages and limitations are presented.

### 4.1 Chemical ligation

Chemical coupling with cyanogen bromide or a water soluble carbodiimide allows synthesis of both 3′-5′- and 2′-5′-phosphodiesters. A terminal monophosphate is required, which is donated by either the 5′- or 3′-substrate, while another substrate supplies the nucleophilic oxygen such as a 3′-OH. The segments to be joined are then hybridized to a complementary template. While the yields for coupling DNA duplexes can be as high as 96%, the efficiency for RNA oligomers is much lower at 4–16% [95–96]. Unfortunately, this chemical method is also prone to side reactions of base functional groups with the coupling reagents, and the reaction can be exceedingly slow [96]. Accordingly, chemical ligation methods are not widely used for synthesis of 3′-5′ RNA phosphodiester linkages. Synthesis of RNA lariats that have 2′-5′ phosphodiester linkages can be slightly more efficient if the water soluble 1-ethyl-3-[3-dimethylaminopropyl]carbodiimide (EDC) reagent is used [97]. In one case, a 22 nt RNA lariat containing a rU branch point was synthesized in 51% yield after a 6 day coupling reaction [97]. The chemical method for RNA ligation is therefore currently very limited.

### 4.2 DNA splinted T4 DNA ligase based ligation

**Figure 9.**
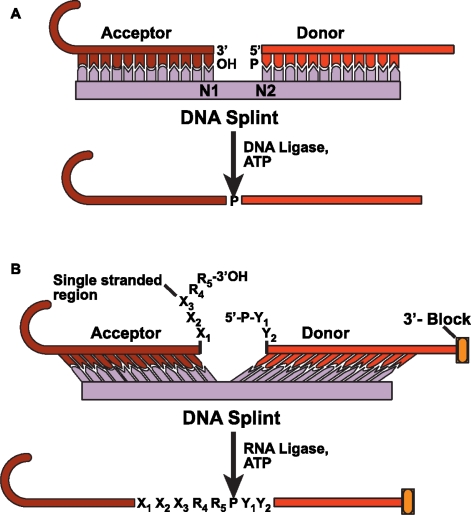
3′-5′-RNA ligation using DNA splints mediated by (A) DNA and (B) RNA ligase.

One of the most widely used methods for ligating RNA molecules employs T4 DNA ligase [98]. DNA ligase in the presence of ATP recognizes a nicked double-stranded substrate by joining one oligonucleotide with a 5′-monophosphate (generally referred to as the donor strand) and another oligonucleotide with a 3′-OH (called the acceptor molecule) (Figure 9A). The RNA substrates used for ligation can be prepared in at least four ways so that the donor substrate begins with a 5′-phosphate and the acceptor terminates in a free 3′-OH. For donor RNA that is chemically synthesized, the 5′-monophosphate can be added during or after the synthesis using T4 polynucleotide kinase and ATP. An *in vitro* transcribed donor RNA can be initiated with GMP or a 5′-XpG-3′ dinucleotide, or the transcribed RNA can be dephosphorylated and then rephosphorylated. For the *in vitro* transcribed RNAs, abortive initiation products are significant, and therefore they must be purified away from the full length donor RNA to prevent competition for the cDNA template. T4 DNA ligase will only ligate full (N) length acceptors. But a significant amount of 3′-end heterogeneity caused by N+1 products can inhibit ligation efficiency by sequestering donor substrates into unproductive hybrid complexes with the cDNA template [98]. The main advantages of this T4 DNA ligase method are: (a) undesired side products such as circularization or oligomerization of the donor RNA are minimized because of strict requirements for double-stranded complexes; (b) the ligase activity is not dependent on the exact sequence at the ligation junction; and (c) most “N+1” T7 RNA polymerase add-ons are effectively excluded. A major disadvantage is that T4 DNA ligase is relatively inefficient at joining RNA molecules and is required at near stoichiometric amounts in ligation reactions. Larger RNAs are needed in milligram quantities for most biophysical studies. But the low yield and the need for large amounts of commercially available, but cost-prohibitive, DNA ligase makes it less likely that DNA ligase will be widely used for large scale RNA production required for structural biology.

### 4.3 DNA splinted T4 RNA based ligation

An alternative method for RNA ligation, used extensively prior to 1992 [99] but now making a resurgence, is to use T4 RNA ligase. This is partly because RNA ligase has some deficiencies that have been overcome only recently [100–102]. RNA ligase, like DNA ligase, requires a donor substrate with a 5′-phosphate, an acceptor with 3′-OH, and ATP (Figure 9B). But unlike DNA ligase, RNA ligase requires single stranded ligation junctions, and therefore using cDNA as a template is difficult. To overcome this limitation, Bain and Switzer [103] designed a DNA splint that could not base-pair to the elements in the immediate region of the splint junction, such that the donor and acceptor molecules were held in close proximity. This created a single-stranded region compatible with T4 RNA ligase specificity. Using this approach, they reported a ligation efficiency of 53%. To achieve higher ligation efficiencies meant overcoming other known difficulties: ligation efficiency improves only if the last two nucleotide sequences of the acceptor terminate in a purine (R_4_ and R_5_ in Figure 9B); uridine at either the penultimate or 3′-terminal position is particularly inefficient for ligation; for donors, at the 5′-terminal position, there is a slight preference for pyrimidines (Y_1_ and Y_2_ in Figure 9B) over purines for improving the ligation efficiency.

Stark and colleagues did exactly that and the following modifications to improve the ligation efficiency to near completion in 30 minutes [101]. First, they protected the donor 3′-OH with a 5′-silyl-2′-acetoxy-ethyl orthoester (2′-ACE) group (3′-block in Figure 9B) used in oligoribonucleotide synthesis. This minimizes the circularization or oligomerization of the donor RNA. Second, they chemically incorporated phosphate on the donor strand to minimize the problem of 5′-end heterogeneity. Third, they designed an optimized linker at the ligation junction comprising 4–8 single-stranded nucleotides on the acceptor RNA (X_1_ X_2_ X_3_ R_3_ R_4_ in Figure 9B) and 1 or 2 nt on the donor RNA (Figure 9B). The splints used in the ligation were designed such that the melting temperature for each half of the splint was at least 40°C and at most 45°C. Using this strategy they successfully synthesized a 128 nt RNA from three synthetic oligonucleotide pieces.

The question still remains: how well do these DNA splinted ligation schemes work in the context of structured RNA elements? Unfortunately very few studies have documented the influence of structure on DNA splinted ligation efficiencies using T4 RNA ligase. Preliminary studies from our own group suggest that even large (~500 nt), structured RNAs can be ligated to obtain yields of ~33% (Dayie KT & Agyeman A, unpublished).

### 4.4 Deoxyribozyme based ligation

**Figure 10.**
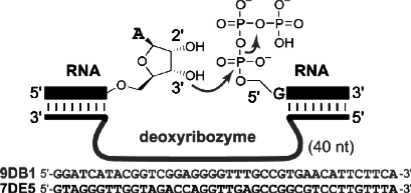
DNAzyme mediated 3′-5′-RNA ligation [105].

Another alternative for the synthesis of native 3′-5′-RNA linkage is to use deoxyribozymes [104–105]. Silverman'sgroup identified two artificial deoxyribozymes using *in vitro* selection experiments. Unlike the T4 protein ligases, the two promising ligases require a 2′, 3′-diol for the acceptor fragment and a 5′-triphosphate functional group for the donor RNA fragment (Figure 10). The two ligases are a Mg^2+^-dependent 9DB1 and a Zn^2+^-dependent 7DES deoxyribozymes [105]. The Mg^2+^-dependent ligase gives the highest ligation yield at 60–70% with a t_1/2_ of ~15 minutes, but it functions at the rather high pH of 9.0 and elevated Mg^2+^ at 37°C. These reaction conditions may promote nonspecific RNA degradation, substantially limiting the usefulness of this ligase for joining large pieces of RNA. Incubation at pH 7.5, while possible, lengthens the t_1/2_ 16 fold, again limiting its usefulness. This DNAzyme has less restrictive sequence requirements at the ligation site, D^↓^RA (where D = A, G, or U; and R = A or G). The Zn^2+^-dependent ligase is less efficient at ligation than 9DB1 (40–50%). Nonetheless it ligates under practical *in vitro* conditions that reduce the potential for RNA degradation. At 23°C, pH 7.5 and 1 mM Zn^2+^, this ligase has a t_1/2_ of 30 minutes. Together, both ligases provide a useful alternative to protein-mediated RNA ligation. In the future, efforts to evolve such DNAzymes to work under more physiological conditions to ligate 3′-5′-bonds of any two RNA sequences will be extremely valuable.

## 5. Tackling sizeable problems in RNA biophysical chemistry

In the post-genomic structural biology era, large size matters: most molecular machines are mega Dalton machines, making traditional NMR techniques inadequate for the task. For these large systems, cryo-electron microscopic reconstructions can provide images of the overall shape but without the requisite molecular details. X-ray crystallography is ideally suited for providing these atomic details, yet is limited to well structured and conformationally homogeneous systems. NMR spectroscopy is ideally positioned to tackle such systems. As discussed above, the problems of overlap and size that plague traditional approaches can be effectively circumvented using these emerging technologies. In particular, modular multi-component biological systems are particularly suited for a divide-and-conquer approach that can take advantage of these technologies. By divide-and-conquer we mean working on multi-component systems that can be broken down into folded “domains” and then probing the labeled components singly or in various combinations with unlabeled components. In this last section, two biological problems that are amenable to this approach are described.

### 5.1 Structural basis for encapsidation of the genome of the Moloney Murine Leukemia Virus

Retroviruses are a family of RNA viruses that cause a number of diseases such as AIDS, neurological disorders, tumors, and leukemias [106]. The structural basis for how retroviruses select and package their genomes will be helpful for designing new antiviral therapies. The Moloney Murine Leukemia Virus (MMLV) is widely used in human gene therapy trials and as a model system to study retroviral assembly and genome encapsidation. It contains a 350-nt fragment called the Ψ-site that directs packaging and dimerization of the viral RNA. Remarkably, a sub-fragment of the Ψ-site containing ~100 nt can direct RNA packaging into virus-like particles, albeit at reduced efficiency. To gain insight into the molecular basis of genome packaging in MMLV, D'Souza and Summers determined, using NMR, the 3D structure of the 101 nt RNA sub-fragment of the Ψ-site in complex with a nucleocapsid domain of the Gag polyproteins important for mediating this interaction [107]. To tackle the largest known NMR RNA structure, the authors had to use a nucleotide-specific isotopic labeling strategy to overcome the extensive chemical shift overlap [61]. These methods are analogous to those described in making labeled NTPs in sections 3.0–3.4 above. With this approach, they were able to distinguish internucleotide from intranucleotide nuclear Overhauser effects (NOE), and obtain valuable distance information to compute a moderate resolution structure [61, 107]. The disadvantage of this approach is that it required preparing eight different isotopically labeled samples and collecting several 2D, 3D, and 4D NMR datasets, making it both labor and instrumentation-intensive [61]. Uses of some of the technologies discussed above are likely to help with getting higher resolution structures of this biologically important complex.

### 5.2 Towards the structural basis for Group II Intron ribozyme catalytic funtion

A large riboncucleoprotein complex called the spliceosome [4] mediates the accurate removal of intervening RNA sequences (introns) from eukaryotic pre-mRNAs [4, 108]. At the heart of this critical biological process lies a simple chemical reaction, which is carried out by nucleophilic attack on the 5′-splice site phosphate by the 2′-hydroxyl of an intronic branch point adenosine to generate a free 5′-exon and an intron-3′-exon. In the following step, the freed 3′-hydroxyl of the 5′-exon attacks the 3′-splice site to release the intron and ligate the 3′-exon to the 5′-exon. Remarkably, a class of RNAs called group II introns (G2I) performs the same chemical reactions as the spliceosome, yet only uses RNA. Because the group II introns share several structural features and identical reaction mechanism with the messenger RNA splicing machinery, they are an important model system for exploring the mechanisms for RNA catalysis. The key question is: what is the molecular basis of catalysis? Determining the 3D architecture of the group II intron is the beginning process to answer this question, and in turn this requires defining an experimentally well-behaved model system. We recently reconstructed a group II intron from *Pylaiella littoralis* into a highly active form by combining an unstructured 22 nt substrate, a 36 nt domain 5 (D5) hairpin, and a 493 nt domains 1 through 3 (D123) [109–112]. This new tripartite ribozyme system functions under low salt conditions attractive for NMR structural studies and has enabled us to determine a very high resolution NMR structure of the catalytic lynchpin, D5 [110]. We have also quantitatively assayed the binding of each component using a fluorescence native gel mobility shift assay we developed in conjunction with fluorescent anisotropy measurements [109, 112]. Parenthetically, this fluorescent method takes advantage of a multicolor fluorescent-based gel assay to directly monitor RNA-RNA interactions, without the need to use hazardous ^32^P labeled probes [109, 112–113]. Using pre-steady state-kinetic experiments, we have shown that the cleavage reaction is pH-dependent, suggesting that the chemical step is rate limiting [109].

Using a divide-and-conquer approach, we have shown that NMR experiments can provide a detailed view of the binding interface between the catalytically important D5 and the large 100 kDa D123 fragment. The regions of D5 that undergo the greatest chemical shift changes correspond to those areas predicted by footprinting experiments [109]. Using NMR relaxation measurements, and newly developed software for RNA dynamic analysis, we have shown that regions of D5 important for catalysis and binding are flexible, suggesting that motion is an important component of catalysis that requires further exploration [111].

In conclusion, using very simple NMR experiments on multidomain structures, wherein one component is preferentially labeled with NMR-active nuclei, one can probe in atomic detail the binding interface of numerous molecular machines [61, 109, 114]. In the case of group II introns, this approach enabled us to identify a key aspect of catalysis: the highly conserved catalytic lynchpin, D5, must undergo large conformational changes to adapt to the rest of the intronic elements to carry out catalysis [109–111]. We anticipate that similar approaches applied to other large macromolecular RNA complexes will provide fundamental insight into biological functions that may not be readily accessible from static X-ray structures.

## References

[1] Gesteland RF, Cech TR, Atkins JF (2006). RNA World.

[2] Korostelev A, Noller HF (2007). The ribosome in focus: new structures bring new insights. Trends Biochem Sci.

[3] Steitz TA (2008). A structural understanding of the dynamic ribosome machine. Nat Rev Mol Cell Biol.

[4] Stark H, Lührmann R (2006). Cryo-electron microscopy of spliceosomal components. Annu Rev Biophys Biomol Struct.

[5] Bessonov S, Anokhina M, Will CL, Urlaub H, Luhrmann R (2008). Isolation of an active step I spliceosome and composition of its RNP core. Nature.

[6] Boisvert FM, van Koningsbruggen S, Navascués J, Lamond AI (2007). The multifunctional nucleolus. Nat Rev Mol Cell Biol.

[7] Wakeman CA, Winkler WC, Dann CE (2007). Structural features of metabolite-sensing riboswitches. Trends Biochem Sci.

[8] Edwards TE, Klein DJ, Ferré-D'Amaré AR (2007). Riboswitches: small-molecule recognition by gene regulatory RNAs. Curr Opin Struct Biol.

[9] Taft RJ, Pheasant M, Mattick JS (2007). The relationship between non-protein-coding DNA and eukaryotic complexity. Bioessays.

[10] Amaral PP, Dinger ME, Mercer TR, Mattick JS (2008). The eukaryotic genome as an RNA machine. Science.

[11] Cromsigt J, van Buuren B, Schleucher J, Wijmenga S (2001). Resonance assignment and structure determination for RNA. Methods Enzymol.

[12] Wijmenga SS, van Buuren BNM (1998). The use of NMR methods for conformational studies of nucleic acids. Prog NMR Spectrosc.

[13] Furtig B, Richter C, Bermel W, Schwalbe H (2004). New NMR experiments for RNA nucleobase resonance assignment and chemical shift analysis of an RNA UUCG tetraloop. J Biomol NMR.

[14] Latham MP, Brown DJ, McCallum SA, Pardi A (2005). NMR methods for studying the structure and dynamics of RNA. Chembiochem.

[15] Tzakos AG, Grace CR, Lukavsky PJ, Riek R (2006). NMR techniques for very large proteins and RNAs in solution. Annu Rev Biophys Biomol Struct.

[16] Golden BL, Kundrot CE (2003). RNA crystallization. J Struct Biol.

[17] Golden BL (2007). Preparation and crystallization of RNA. Methods Mol Biol.

[18] Cate JH, Doudna JA (2000). Solving large RNA structures by X-ray crystallography. Methods Enzymol.

[19] Ke A, Doudna JA (2004). Crystallization of RNA and RNA-protein complexes. Methods.

[20] Scaringe SA (2001). RNA oligonucleotide synthesis via 5′-silyl-2′-orthoester chemistry. Methods.

[21] Krieg PA, Melton DA (1987). *In vitro* RNA synthesis with SP6 RNA polymerase. Methods Enzymol.

[22] Milligan JF, Uhlenbeck OC (1989). Synthesis of small RNAs using T7 RNA polymerase. Methods Enzymol.

[23] Pokrovskaya ID, Gurevich VV (1994). *In vitro* transcription: preparative RNA yields in analytical scale reactions. Anal Biochem.

[24] Studier FW, Rosenberg AH, Dunn JJ, Dubendorff JW (1990). Use of T7 RNA polymerase to direct expression of cloned genes. Methods Enzymol.

[25] Pleiss JA, Derrick ML, Uhlenbeck OC (1998). T7 RNA polymerase produces 5′ end heterogeneity during *in vitro* transcription from certain templates. RNA.

[26] Helm M, Brulé H, Giegé R, Florentz C (1999). More mistakes by T7 RNA polymerase at the 5′ ends of *in vitro*-transcribed RNAs. RNA.

[27] Krupp G (1988). RNA synthesis: strategies for the use of bacteriophage RNA polymerases. Gene.

[28] Wyatt JR, Chastain M, Puglisi JD (1991). Synthesis and purification of large amounts of RNA oligonucleotides. Biotechniques.

[29] Puglisi JD, Wyatt JR (1995). Biochemical and NMR studies of RNA conformation with an emphasis on RNA pseudoknots. Methods Enzymol.

[30] Grosshans CA, Cech TR (1991). A hammerhead ribozyme allows synthesis of a new form of the Tetrahymena ribozyme homogeneous in length with a 3′ end blocked for transesterification. Nucleic Acids Res.

[31] Ferré-D'Amaré AR, Doudna JA (1996). Use of cis- and trans-ribozymes to remove 5′ and 3′ heterogeneities from milligrams of *in vitro* transcribed RNA. Nucleic Acids Res.

[32] Moran S, Ren RX, Sheils CJ, Rumney S, Kool ET (1996). Non-hydrogen bonding ‘terminator’ nucleosides increase the 3′-end homogeneity of enzymatic RNA and DNA synthesis. Nucleic Acids Res.

[33] Kao C, Zheng M, Rüdisser S (1999). A simple and efficient method to reduce nontemplated nucleotide addition at the 3 terminus of RNAs transcribed by T7 RNA polymerase. RNA.

[34] Coleman TM, Wang G, Huang F (2004). Superior 5′ homogeneity of RNA from ATP-initiated transcription under the T7 phi 2.5 promoter. Nucleic Acids Res.

[35] Dayie KT (2005). Resolution enhanced homonuclear carbon decoupled triple resonance experiments for unambiguous RNA structural characterization. J Biomol NMR.

[36] Ponchon L, Dardel F (2007). Recombinant RNA technology: the tRNA scaffold. Nat Methods.

[37] Wincott F, DiRenzo A, Shaffer C, Grimm S, Tracz D, Workman C, Sweedler D, Gonzalez C, Scaringe S, Usman N (1995). Synthesis, deprotection, analysis and purification of RNA and ribozymes. Nucleic Acids Res.

[38] Anderson AC, Scaringe SA, Earp BE, Frederick CA (1996). HPLC purification of RNA for crystallography and NMR. RNA.

[39] Shields TP, Mollova E, Ste Marie L, Hansen MR, Pardi A (1999). High-performance liquid chromatography purification of homogenous-length RNA produced by trans cleavage with a hammerhead ribozyme. RNA.

[40] Sich C, Ohlenschlager O, Ramachandran R, Gorlach M, Brown LR (1997). Structure of an RNA hairpin loop with a 5′-CGUUUCG-3′ loop motif by heteronuclear NMR spectroscopy and distance geometry. Biochemistry.

[41] Azarani A, Hecker KH (2001). RNA analysis by ion-pair reversed-phase high performance liquid chromatography. Nucleic Acids Res.

[42] Dickman MJ, Conroy MJ, Grasby JA, Hornby DP (2002). RNA footprinting analysis using ion pair reverse phase liquid chromatography. RNA.

[43] Nikonowicz EP, Sirr A, Legault P, Jucker FM, Baer LM, Pardi A (1992). Preparation of 13C and 15N labelled RNAs for heteronuclear multi-dimensional NMR studies. Nucleic Acids Res.

[44] Batey RT, Inada M, Kujawinski E, Puglisi JD, Williamson JR (1992). Preparation of isotopically labeled ribonucleotides for multidimensional NMR spectroscopy of RNA. Nucleic Acids Res.

[45] Cheong HK, Hwang E, Lee C, Choi BS, Cheong C (2004). Rapid preparation of RNA samples for NMR spectroscopy and X-ray crystallography. Nucleic Acids Res.

[46] Kieft JS, Batey RT (2004). A general method for rapid and nondenaturing purification of RNAs. RNA.

[47] Batey RT, Kieft JS (2007). Improved native affinity purification of RNA. RNA.

[48] Crowe J, Döbeli H, Gentz R, Hochuli E, Stüber D, Henco K (1994). 6xHis-Ni-NTA chromatography as a superior technique in recombinant protein expression/purification. Methods Mol Biol.

[49] Winkler WC, Nahvi A, Roth A, Collins JA, Breaker RR (2004). Control of gene expression by a natural metabolite-responsive ribozyme. Nature.

[50] Hampel KJ, Tinsley MM (2006). Evidence for preorganization of the glmS ribozyme ligand binding pocket. Biochemistry.

[51] Lukavsky PJ, Puglisi JD (2004). Large-scale preparation and purification of polyacrylamide-free RNA oligonucleotides. RNA.

[52] Kim I, McKenna SA, Viani-Puglisi E, Puglisi JD (2007). Rapid purification of RNAs using fast performance liquid chromatography (FPLC). RNA.

[53] McKenna SA, Kim I, Puglisi EV, Lindhout DA, Aitken CE, Marshall RA, Puglisi JD (2007). Purification and characterization of transcribed RNAs using gel filtration chromatography. Nat Protoc.

[54] Michnicka MJ, Harper JW, King GC (1993). Selective isotopic enrichment of synthetic RNA: application to the HIV-1 TAR element. Biochemistry.

[55] Hines JV, Landry SM, Varani G, Tinoco I (1994). Carbon-Proton Scalar Couplings in RNA: 3D Heteronuclear and 2D Isotope-Edited NMR of a ^13^C-Labeled Extra-stable Hairpin. J Am Chem Soc.

[56] McIntosh LP, Dahlquist FW (1990). Biosynthetic incorporation of 15N and 13C for assignment and interpretation of nuclear magnetic resonance spectra of proteins. Q Rev Biophys.

[57] Dieckmann T, Feigon J (1997). Assignment methodology for larger RNA oligonucleotides: application to an ATP-binding RNA aptamer. J Biomol NMR.

[58] Pardi A (1995). Multidimensional heteronuclear NMR experiments for structure determination of isotopically labeled RNA. Methods Enzymol.

[59] Latham MP, Brown DJ, McCallum SA, Pardi A (2005). NMR methods for studying the structure and dynamics of RNA. Chembiochem.

[60] Batey RT, Cloutier N, Mao H, Williamson JR (1996). Improved large scale culture of Methylophilus methylotrophus for 13C/15N labeling and random fractional deuteration of ribonucleotides. Nucleic Acids Res.

[61] D'Souza V, Dey A, Habib D, Summers MF (2004). NMR structure of the 101-nucleotide core encapsidation signal of the Moloney murine leukemia virus. J Mol Biol.

[62] Peterson RD, Theimer CA, Wu H, Feigon J (2004). New applications of 2D filtered/edited NOESY for assignment and structure elucidation of RNA and RNA-protein complexes. J Biomol NMR.

[63] Hoffman DW, Holland JA (1995). Preparation of carbon-13 labeled ribonucleotides using acetate as an isotope source. Nucleic Acids Res.

[64] Johnson JE, Julien KR, Hoogstraten CG (2006). Alternate-site isotopic labeling of ribonucleotides for NMR studies of ribose conformational dynamics in RNA. J Biomol NMR.

[65] Hoogstraten CG, Johnson JE (2008). Metabolic labeling: Taking advantage of bacterial pathways to prepare spectroscopically useful isotope patterns in proteins and nucleic acids. Concepts in Magnetic Resonance Part A.

[66] Brutscher B, Boisbouvier J, Kupce E, Tisné C, Dardel F, Marion D, Simorre JP (2001). Base-type-selective high-resolution ^13^C edited NOESY for sequential assignment of large RNAs. J Biomol NMR.

[67] Markley JL, Putter I, Jardetzky O (1968). High-resolution nuclear magnetic resonance spectra of selectively deuterated staphylococcal nuclease. Science.

[68] LeMaster DM (1990). Deuterium labelling in NMR structural analysis of larger proteins. Q Rev Biophys.

[69] Markus MA, Dayie KT, Matsudaira P, Wagner G (1994). Effect of deuteration on the amide proton relaxation rates in proteins. Heteronuclear NMR experiments on villin 14T. J Magn Reson B.

[70] Tolbert TJ, Williamson JR (1997). Preparation of Specifically Deuterated and ^13^C-Labeled RNA for NMR Studies Using Enzymatic Synthesis. J Am Chem Soc.

[71] Dayie KT, Tolbert TJ, Williamson JR (1998). 3D C(CC)H TOCSY experiment for assigning protons and carbons in uniformly ^13^C- and selectively ^2^H-labeled RNA. J Magn Reson.

[72] Gardner KH, Kay LE (1998). The use of ^2^H, ^13^C, ^15^N multidimensional NMR to study the structure and dynamics of proteins. Annu Rev Biophys Biomol Struct.

[73] Nikonowicz EP (2001). Preparation and use of ^2^H-labeled RNA oligonucleotides in nuclear magnetic resonance studies. Methods Enzymol.

[74] Scott LG, Tolbert TJ, Williamson JR (2000). Preparation of specifically ^2^H- and ^13^C-labeled ribonucleotides. Methods Enzymol.

[75] Davis JH, Tonelli M, Scott LG, Jaeger L, Williamson JR, Butcher SE (2005). RNA helical packing in solution: NMR structure of a 30 kDa GAAA tetraloop-receptor complex. J Mol Biol.

[76] Tolbert TJ, Williamson JR (1996). Preparation of Specifically Deuterated RNA for NMR Studies Using a Combination of Chemical and Enzymatic Synthesis. J Am Chem Soc.

[77] Vallurupalli P, Scott L, Hennig M, Williamson JR, Kay LE (2006). New RNA labeling methods offer dramatic sensitivity enhancements in ^2^H NMR relaxation spectra. J Am Chem Soc.

[78] Hirschbein BL, Mazenod FP, Whitesides GM (1982). Synthesis of phosphoenolypyruvate and its use in ATP cofactor regeneration. J Org Chem.

[79] Simon ES, Grabowski S, Whitesides GM (1989). Preparation of phosphoenolpyruvate from D-(−)-3-phosphoglyceric acid for use in regeneration of ATP. J Am Chem Soc.

[80] Rising KA, Schramm VL (1994). Enzymic Synthesis of NAD+ with the Specific Incorporation of Atomic Labels. J Am Chem Soc.

[81] Scott LG, Geierstanger BH, Williamson JR, Hennig M (2004). Enzymatic synthesis and ^19^F NMR studies of 2-fluoroadenine-substituted RNA. J Am Chem Soc.

[82] Hennig M, Scott LG, Sperling E, Bermel W, Williamson JR (2007). Synthesis of 5-fluoropyrimidine nucleotides as sensitive NMR probes of RNA structure. J Am Chem Soc.

[83] Toth EA (2007). Molecular replacement. Methods Mol Biol.

[84] Golden BL, Gooding AR, Podell ER, Cech TR (1996). X-ray crystallography of large RNAs: heavy-atom derivatives by RNA engineering. RNA.

[85] Golden BL (2000). Heavy atom derivatives of RNA. Methods Enzymol.

[86] Wedekind JE, McKay DB (2000). Purification, crystallization, and X-ray diffraction analysis of small ribozymes. Methods Enzymol.

[87] Correll CC, Freeborn B, Moore PB, Steitz TA (1997). Use of chemically modified nucleotides to determine a 62-nucleotide RNA crystal structure: a survey of phosphorothioates, Br, Pt and Hg. J Biomol Struct Dyn.

[88] Baugh C, Grate D, Wilson C (2000). 2.8 A crystal structure of the malachite green aptamer. J Mol Biol.

[89] Martick M, Scott WG (2006). Tertiary contacts distant from the active site prime a ribozyme for catalysis. Cell.

[90] Ferre-D'Amare AR, Doudna JA (2000). Crystallization and structure determination of a hepatitis delta virus ribozyme: use of the RNA-binding protein U1A as a crystallization module. J Mol Biol.

[91] Ferre-D'Amare AR, Zhou K, Doudna JA (1998). Crystal structure of a hepatitis delta virus ribozyme. Nature.

[92] Sheng J, Huang Z (2008). Selenium Derivatization of Nucleic Acids for Phase and Structure Determination in Nucleic Acid X-ray Crystallography. Int J Mol Sci.

[93] Brandt G, Carrasco N, Huang Z (2006). Efficient substrate cleavage catalyzed by hammerhead ribozymes derivatized with selenium for X-ray crystallography. Biochemistry.

[94] Keel AY, Rambo RP, Batey RT, Kieft JS (2007). A general strategy to solve the phase problem in RNA crystallography. Structure.

[95] Sokolova NI, Ashirbekova DT, Dolinnaya NG, Shabarova ZA (1988). Chemical reactions within DNA duplexes. Cyanogen bromide as an effective oligodeoxyribonucleotide coupling agent. FEBS Lett.

[96] Dolinnaya NG, Sokolova NI, Ashirbekova DT, Shabarova ZA (1991). The use of BrCN for assembling modified DNA duplexes and DNA-RNA hybrids; comparison with water-soluble carbodiimide. Nucleic Acids Res.

[97] Mitra D, Damha MJ (2007). A novel approach to the synthesis of DNA and RNA lariats. J Org Chem.

[98] Moore MJ, Query CC (2000). Joining of RNAs by splinted ligation. Methods Enzymol.

[99] Romaniuk PJ, Uhlenbeck OC (1983). Joining of RNA molecules with RNA ligase. Methods Enzymol.

[100] Tzakos AG, Easton LE, Lukavsky PJ (2007). Preparation of large RNA oligonucleotides with complementary isotope-labeled segments for NMR structural studies. Nat Protoc.

[101] Stark MR, Pleiss JA, Deras M, Scaringe SA, Rader SD (2006). An RNA ligase-mediated method for the efficient creation of large, synthetic RNAs. RNA.

[102] Kim I, Lukavsky PJ, Puglisi JD (2002). NMR study of 100 kDa HCV IRES RNA using segmental isotope labeling. J Am Chem Soc.

[103] Bain JD, Switzer C (1992). Regioselective ligation of oligoribonucleotides using DNA splints. Nucleic Acids Res.

[104] Höbartner C, Silverman SK (2007). Recent advances in DNA catalysis. Biopolymers.

[105] Purtha WE, Coppins RL, Smalley MK, Silverman SK (2005). General deoxyribozyme-catalyzed synthesis of native 3′-5′ RNA linkages. J Am Chem Soc.

[106] D'Souza V, Summers MF (2005). How retroviruses select their genomes. Nat Rev Microbiol.

[107] D'Souza V, Summers MF (2004). Structural basis for packaging the dimeric genome of Moloney murine leukaemia virus. Nature.

[108] Pyle AM, Lambowitz AM, Gesteland RF, Cech TR, Atkins JF (2006). Group II introns: Ribozymes that splice RNA and invade DNA. RNA World.

[109] Gumbs OH, Padgett RA, Dayie KT (2006). Fluorescence and solution NMR study of the active site of a 160-kDa group II intron ribozyme. RNA.

[110] Seetharaman M, Eldho NV, Padgett RA, Dayie KT (2006). Structure of a self-splicing group II intron catalytic effector domain 5: parallels with spliceosomal U6 RNA. RNA.

[111] Eldho NV, Dayie KT (2007). Internal bulge and tetraloop of the catalytic domain 5 of a group II intron ribozyme are flexible: implications for catalysis. J Mol Biol.

[112] Dayie KT, Gumbs OH, Eldho NV, Seetharaman M, Thompson M (2007). In-gel fluorescence probing of RNA-RNA interactions. Anal Biochem.

[113] Ying BW, Fourmy D, Yoshizawa S (2007). Substitution of the use of radioactivity by fluorescence for biochemical studies of RNA. RNA.

[114] Szymczyna BR, Gan L, Johnson JE, Williamson JR (2007). Solution NMR studies of the maturation intermediates of a 13 MDa viral capsid. J Am Chem Soc.

[115] Toor N, Keating KS, Taylor SD, Pyle AM (2008). Crystal structure of a self-spliced group II intron. Science.

[116] Dayie KT, Padgett RA (2008). A glimpse into the active site of the Group II intron and maybe the spliceosome too. RNA.

